# Supplementary material for: MetaRibo-Seq measures translation in microbiomes
Source: Nat Commun. 2020 Jun 29;11:3268. doi: 10.1038/s41467-020-17081-z (PMC7324362; doi:10.1038/s41467-020-17081-z)
Supplement: Supplementary file 10 — Supplementary Data 7 [file 41467_2020_17081_MOESM10_ESM.zip › File2/Confidence_VeryHigh_Taxonomy/320837_out.krona.html]

Javascript must be enabled to view this page.

members
magnitude
magnitudeUnassigned
count
unassigned
taxon
rank

320837\_out

14


SRS014979\_contig\_number\_contig-100\_30717.30718
1

13
2
superkingdom

13
phylum
1239


SRS011084\_contig\_number\_27337SRS011239\_contig\_number\_24649SRS013098\_contig\_number\_13669SRS013521\_contig\_number\_2698SRS018351\_contig\_number\_1805SRS021484\_contig\_number\_3439
1263003
species
6

7
class
186801

7
186802
order

5

SRS012969\_contig\_number\_20031SRS015694\_contig\_number\_16332SRS021948\_contig\_number\_2574SRS022071\_contig\_number\_23621SRS022524\_contig\_number\_4584
species
172733

2
216572
family

2
genus
459786

species
1262910

SRS013687\_contig\_number\_4370
1

1
1235797
species

SRS1054691\_contig\_number\_contig-100\_9266.9267
